# Improved YOLO v5s-based detection method for external defects in potato

**DOI:** 10.3389/fpls.2025.1527508

**Published:** 2025-02-18

**Authors:** XiLong Li, FeiYun Wang, Yalin Guo, Yijun Liu, HuangZhen Lv, Fankui Zeng, Chengxu Lv

**Affiliations:** ^1^ Chinese Academy of Agricultural Mechanization Sciences Croup Co., Ltd., Beijing, China; ^2^ Key Laboratory of Agricultural Products Processing Equipment in the Ministry of Agriculture and Rural Affairs, Beijing, China; ^3^ China National Packaging and Food Machinery Co., Ltd., Beijing, China; ^4^ Research Center for Natural Medicine and Chemical Metrology, Lanzhou Institute of Chemical Physics, Chinese Academy of Sciences, Lanzhou, China

**Keywords:** potato, external defect, object detection, YOLO v5s, deep learning

## Abstract

Currently, potato defect sorting primarily relies on manual labor, which is not only inefficient but also prone to bias. Although automated sorting systems offer a potential solution by integrating potato detection models, real-time performance remains challenging due to the need to balance high accuracy and speed under limited resources. This study presents an enhanced version of the YOLO v5s model, named YOLO v5s-ours, specifically designed for real-time detection of potato defects. By integrating Coordinate Attention (CA), Adaptive Spatial Feature Fusion (ASFF), and Atrous Spatial Pyramid Pooling (ASPP) modules, the model significantly improves detection accuracy while maintaining computational efficiency. The model achieved 82.0% precision, 86.6% recall, 84.3% F1-Score and 85.1% mean average precision across six categories — healthy, greening, sprouting, scab, mechanical damage, and rot — marking improvements of 24.6%, 10.5%, 19.4%, and 13.7%, respectively, over the baseline model. Although memory usage increased from 13.7 MB to 23.3 MB and frame rate slightly decreased to 30.7 fps, the accuracy gains ensure the model’s suitability for practical applications. The research provides significant support for the development of automated potato sorting systems, advancing agricultural efficiency, particularly in real-time applications, by overcoming the limitations of traditional methods.

## 1 Introduction

Potatoes rank as the fourth most cultivated food crop globally, following rice, wheat, and corn (Cui et al., 2020). With China leading in production area and annual output (Luo et al., 2022). Beyond their nutritional and medicinal value, potatoes are a key raw material for various industries, including plastics, paper, and chemicals, largely due to their starch content. However, defects such as greening, sprouting, scabbing, mechanical damage, and rot can arise during harvesting, transportation, and storage, which significantly reduce their quality and market value.

Currently, potato defect detection primarily relies on manual inspection, which is time-consuming, labor-intensive, and subject to human error. This reliance on manual methods has limited advancements in automated potato processing. Researchers have increasingly turned to machine vision technologies to enhance defect detection efficiency. For instance, Li et al. (2018) used color-saturation and three-dimensional geometric features to identify potato sprout eyes, achieving a recognition rate of 91.48%. Similarly, Lv et al. (2021) employed Gabor features for potato image filtering, reaching a 93.4% recognition rate for sprouting. Despite these successes, traditional image- processing methods have limited ability to simultaneously detect multiple defect types, which reduces their practical applicability.

Deep learning techniques have advanced the field by enabling automatic learning of low- and high-level features in images, and object detection models are now widely applied in agricultural inspections (He et al., 2022; Li et al., 2023; Senthil et al., 2023; Wang et al., 2023). Existing approaches can generally be divided into two categories: two-stage detectors, represented by Faster R-CNN (Ren et al., 2017), and single-stage detectors, such as YOLO (Redmon and Farhadi, 2018; Bochkovskiy et al., 2020; Ge et al., 2021; Zhu et al., 2021) and SSD (Zhao et al., 2019). While two-stage detectors excel in accuracy, single-stage detectors like YOLO offer a better balance between detection speed and accuracy, which is critical for real-time applications. Recent YOLO-based studies in potato inspection have yielded positive results. For example, Zhang et al. (2023) improved the YOLO v5 model by incorporating an attention mechanism for detecting potato seed eyes. Shi et al. (2022) enhanced YOLO v3 by expanding the dataset to include occluded seed eyes, mechanical damage, and impurities. Zhang et al. (2021) combined Mobilenet V3 and YOLO v4 networks to accurately and efficiently detect potato damage during the harvesting process. Fu et al. (2021) applied YOLO v4 to detect various surface defects, while Yang et al. (2022) combined YOLO v3 tiny with the Res2Net module to detect issues like sprouting and damage.

These YOLO models have made strides in potato defect detection, but the simultaneous detection of multiple defects is still relatively rare. The high similarity among certain potato defect types and the potential loss of feature information during downsampling can reduce model accuracy. Standard YOLO models are not optimized to address these issues fully. Therefore, this study proposes an improved YOLO v5s-based model for detecting external defects in potatoes. By incorporating Coordinate Attention (CA) to capture essential features and combining Adaptively Spatial Feature Fusion (ASFF) with Atrous Spatial Pyramid Pooling (ASPP) to enhance multi-scale features fusion, our model aims to achieve higher accuracy without compromising speed. This enhanced detection method inherits the detection of multiple single defects and holds significant potential in advancing automated potato sorting systems and improving agricultural processing efficiency.

## 2 Materials and methods

### 2.1 Model construction

#### 2.1.1 Network structure of the improved YOLO v5s

YOLO is an end-to-end object detection model that processes images directly to identify classes and bounding boxes, achieving high speed and accuracy. Unlike region-based models that require many candidate boxes for classification and localization, YOLO offers greater efficiency, especially for real-time applications. YOLO v5 builds on YOLO v4, with improvements in model structure, network architecture, and loss functions. This study employs YOLO v5s, a lighter model ideal for real-time defect detection in agricultural applications. The network comprises four parts: Input, Backbone, Neck, and Prediction.

The Input section applies Mosaic data augmentation, adaptive anchor box computation, and image scaling to enrich the background, optimize anchor boxes, and standardize image sizes. The Backbone uses CBS (Conv2d + Batch Normalization + SiLU), Bottleneck Cross Stage Partial Connections (CSP), and a serial Spatial Pyramid Pooling - Fast (SPPF) (instead of parallel Spatial Pyramid Pooling (SPP)), which reduces computational load and enhances detection speed without sacrificing accuracy. The Neck network, with its Feature Pyramid Networks(FPN) + Path Aggregation Network(PANet) structure, enhances classification and positional information, with the FPN layer reinforcing semantic information from top to bottom and the PAN layer adding positional data from bottom to top. Finally, the Prediction section outputs feature maps at three scales (80×80, 40×40, and 20×20), to detect small, medium, and large targets, which boosts the model’s accuracy and real-time performance in detecting external potato defects.

**Figure 1:**
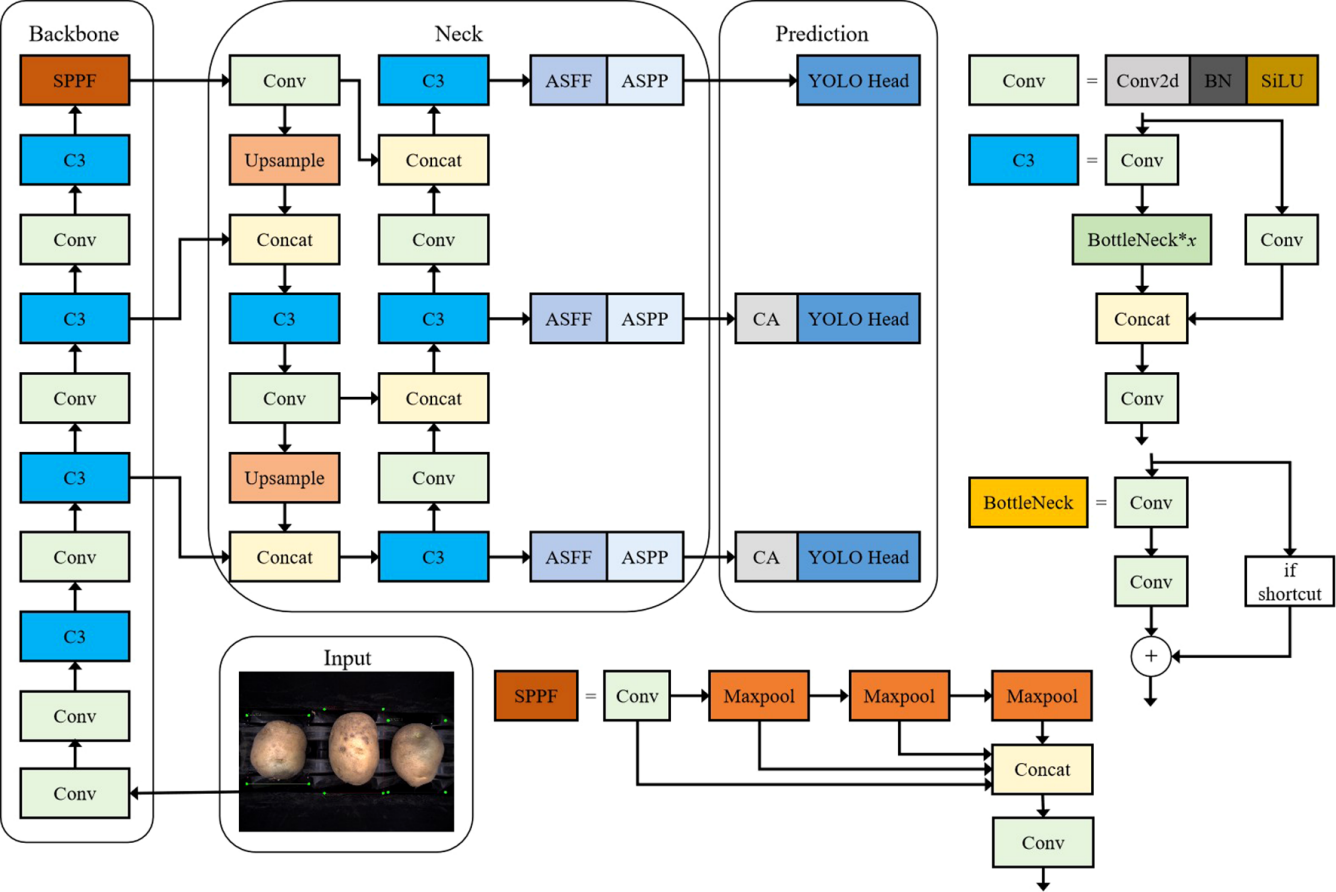
Improved network architecture for YOLO v5s.

This study addresses the challenge of detecting similar potato defects against complex background by integrating the CA mechanism at the front of the detection head, allowing for precise localization. Additionally, the ASFF algorithm and ASPP algorithm are incorporated into the PAN structure to facilitate multi-scale feature fusion and expand the receptive field, which improves feature utilization. Finally, the Alpha-IoU loss function is employed to further enhance detection accuracy. **Figure 1** illustrates the architecture of this improved YOLO v5s model, designed specifically for detecting external defects in potatoes.

#### 2.1.2 Coordinate attention

**Figure 2:**
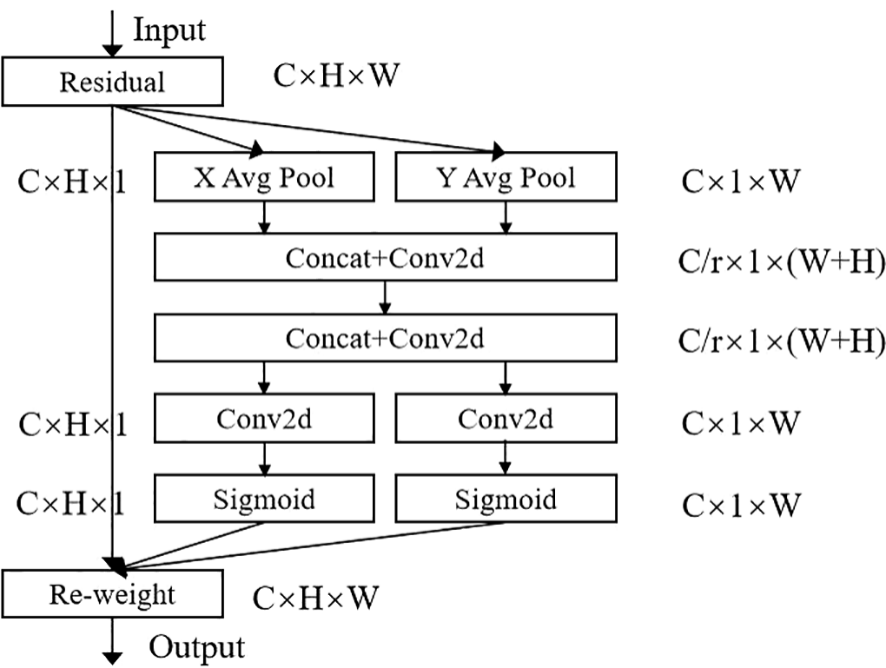
Coordinate attention module structure.

To capture key defect features from complex visual information and accurately distinguish between defects with similar characteristics, this study integrates the CA mechanism. The CA module, illustrated in **Figure 2**, is designed to enable precise localization of defect targets. It consists of two main components: Coordinate Information Embedding (CIE) and Coordinate Attention Generation (CAG). Together, these components incorporate both channel relationships and spatial positional information. By capturing precise positional cues and encoding channel relationships along with long-range dependencies, the CA module substantially improves detection accuracy.

The Coordinate Information Embedding (CIE) component, illustrated in **Figure 2**, includes the X Avg Pool and Y Avg Pool sections. This component enable the attention module to capture long-range spatial dependencies with precise positional information by decomposing global pooling into two one-dimensional feature encoding operations. For each input X, pooling kernels with dimensions (H, 1) and (1, W) are applied to encode each channel along the horizontal and vertical directions, respectively. Consequently, the output for the *c*-th channel at height *h* is expressed by Equation 1.


(1) $$z_{c}^{h}(h)=\frac{1}{W}\sum_{0\leq i\leq W}x_{c}(h,i)$$

The output representation for the *c^th^* channel at width *w* is provided in Equation 2.


(2) $$z_{c}^{w}(h)=\frac{1}{H}\sum_{0\leq j\leq W}x_{c}(j,w)$$

The pooling operations (in Equations 1, 2) break down the global information into two spatially aware components—one focused on the vertical (H) and the other on the horizontal (W) direction. This division helps the model maintain positional accuracy in one direction while capturing long-range dependencies in the other. This is especially useful for detecting defects with varying shapes and orientations, like those on potatoes, where spatial relationships are critical.

The Coordinate Attention Generation stage corresponds to the remaining part of the diagram. In this stage, the two generated feature maps (The data representation after processing by the convolutional neural network) are first transformed and concatenated. Dimensionality reduction is then applied using a 1 × 1 convolutional kernel and an activation function, resulting in the final feature map, as represented in Equation 3.


(3) $$f=\delta (F_{1}([z^{h},z^{w}]))$$

Here, *δ* denotes the concatenation operation, *F_1_* represents the 1×1 convolution operation, *z^h^* is the output feature map for the channel at height *h*, and *z^w^* is the output feature map of the channel at width *w*.

Along the spatial dimension, a split operation divides the feature map into two parts: $f^{h}\in R^{C/r\times H\times 1}$ and $f^{w}\in R^{C/r\times 1\times W}$ . Next, an ascending dimension operation is applied using a 1×1 convolution, followed by a sigmoid activation function to generate the attention vectors $g^{h}\in R^{C\times H\times 1}$ and $g^{w}\in R^{C\times 1\times W}$ , as shown in Equations 4 and 5.


(4) $$g^{h}=\sigma (F_{h}(f^{h}))$$

(5) $$g^{w}=\sigma (F_{w}(f^{w}))$$

In this context, *σ* represents the sigmoid activation function, while *F_h_* and *F_w_* denote the 1×1 convolution operation along the *X* and *Y* directions, respectively. The terms *f^h^* and *f^w^* refer to the output feature maps from the split operation along the *X* and *Y* directions. Using *g^h^* and *g^w^* as attention weights, the final output of the CA module is provided in Equation 6.


(6) $$y_{c}(i,j)=x_{c}(i,j)\times g_{c}^{h}(i)\times g_{c}^{w}(j)$$

Where *x_c_(i,j)* denotes the original feature map, $g_{c}^{h}(i)$ and $g_{c}^{w}(j)$ denote the attention weights in the X and Y directions, and *y_c_(i,j)* denotes the output feature map.

#### 2.1.3 Adaptive spatial feature fusion and Atrous Spatial Pyramid Pooling

To improve the fusion capability of defect feature maps obtained via downsampling, this study incorporates ASFF within the PANet structure of the YOLO v5s algorithm. The ASFF mechanism facilitates weighted fusion at each layer of the FPN structure, with fusion weights dynamically generated from the outputs of the convolutional feature layers. These weights are learnable through gradient backpropagation, allowing for adaptive adjustments throughout the fusion process.

**Figure 3:**
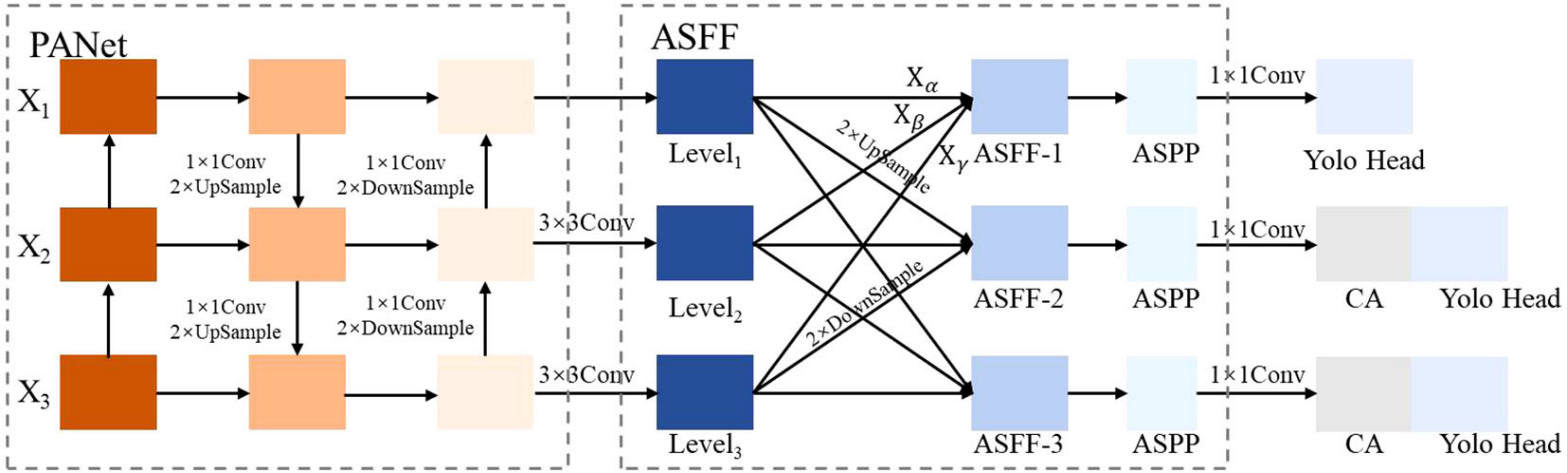
Incorporating ASFF's PANet structure.

After integrating the ASFF mechanism, an ASPP module is added, which applies dilated convolutions at multiple sampling rates to effectively expand the receptive field and enhance the network’s feature extraction capabilities. The proposed PANet structure, incorporating the ASFF and ASPP modules, is shown in **Figure 3**.

Feature maps *X*_1_, *X*_2_ and *X*_3_ are extracted from the YOLO v5s backbone network and processed through the PANet structure to produce Level_1_, Level_2_ and Level_3_. To ensure dimensional consistency with Level_1_, Level_2_ and Level_3_ are downsampled at spatial location (*i*, *j*), resulting in a feature vector of $x_{ij}^{n\to l}$ , which has the same dimensions as Level_1_ and is multiplied by the respective weight coefficient matrices *α*, *β*, and *γ*, followed by summation to yield ASFF-L, which is computed as shown in Equation 7.


(7) $$y_{ij}^{l}=\alpha_{ij}^{l}\cdot x_{ij}^{1\to l}+\beta_{ij}^{l}\cdot x_{ij}^{2\to l}+\gamma_{ij}^{l}\cdot x_{ij}^{3\to l}$$

Equation 7 satisfies constraints $\alpha_{ij}^{l}+\beta_{ij}^{l}+\gamma_{ij}^{l}=1$ and $\alpha_{ij}^{l},\beta_{ij}^{l},\gamma_{ij}^{l}\in [0,1]$ . $\alpha_{ij}^{l}$ are calculated by *softmax* function as shown in Equation 8.


(8) $$\alpha_{ij}^{l}=\frac{e^{\lambda_{\alpha_{ij}}^{l}}}{e^{\lambda_{\alpha_{ij}}^{l}}+e^{\lambda_{\beta_{ij}}^{l}}+e^{\lambda_{\gamma_{ij}}^{l}}}$$

Where the coefficients *λ* are obtained from $x^{1\to l}$ , $x^{2\to l}$ , and $x^{3\to l}$ after a 1×1 convolution operation.

**Figure 4:**
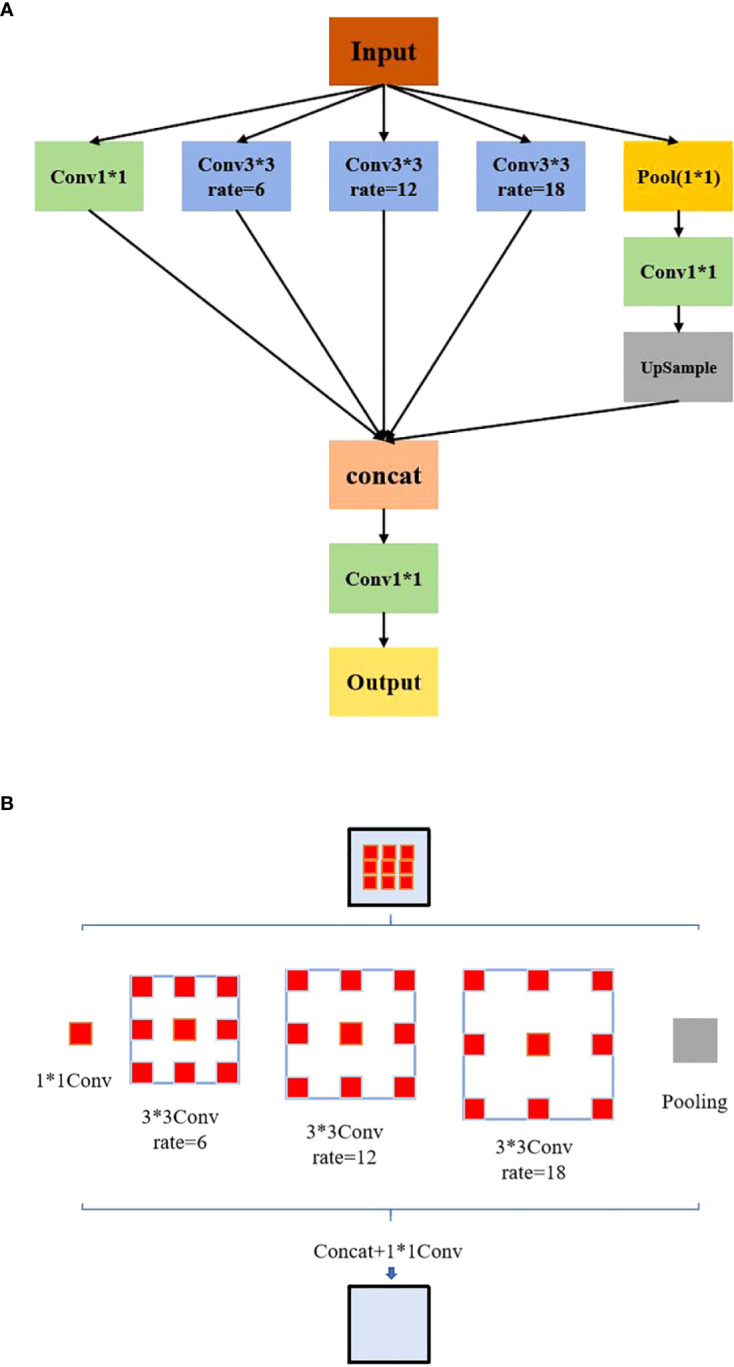
ASPP: **(A)** Structural diagram. **(B)** Module effect diagram.

Following the adjustment of weight coefficients by the ASFF algorithm to optimize multi-scale feature fusion, the ASPP module is applied to further expand the receptive field and improve the utilization of feature information. Through its multi-branch and atrous convolution architecture, the ASPP module enhances the model’s ability to detect and recognize diverse potatoes defects. The ASPP module comprises a 1×1 convolution, a Pooling Pyramid with dilation rates of 6, 12, and 18 (utilizing 3×3 atrous convolution), and ASPP Pooling. Dilation rate is the distance between elements in the convolution kernel. The ASPP structure is shown in **Figure 4A**. The adaptive dilation rates in each layer of the pooling pyramid enable variable receptive fields, allowing the model to capture multi-scale feature information effectively through different rates of expansion and fillings. The effect diagram of the ASPP module is shown in **Figure 4B**.

#### 2.1.4 Alpha-IoU loss function

The prediction component of the YOLO v5s model includes a loss function and a non-maximal suppression mechanism. The loss function quantifies the degree of overlap between the true and predicted bounding boxes. Non-maximal suppression is employed in the post-processing stage of target detection, where it screens multi candidate boxes by suppressing non-maximal values, identifying local maxima, removing redundant boxes, and yielding the final prediction results.

In the standard YOLO v5s model, the GIoU loss function is used, but it has limitations with relatively fixed predictions, as it cannot adaptively adjust loss and gradient weights for targets with high or low IoU values. To address this, we introduce the weight coefficient *α* and replace GIoU with Alpha-IoU as the bounding box loss function. This modification enhances the model’s robustness, as defined in Equations 9 and 10.


(9) $$I^{\prime }=I^{\alpha }-\frac{\rho^{2\alpha }(b,b^{gt})}{d^{2\alpha }}-{\left(\beta \gamma \right)}^{\alpha }$$

(10) $$I=1-I^{\prime }$$

In this context, *I*′ represents the Alpha-IoU value, while *I* denotes the IoU value, the term *ρ^2α^(b, b^gt^)* refers to the Euclidean distance between the centroid *b* of the prediction frame and the centroid *b^gt^* of the real frame. Additionally, *d* represents the diagonal length of the smallest closed region containing the prediction frame and the real frame. The parameter *β* serves as the parameter of trade-off, and *γ* denotes the aspect ratio of the measurement frame.

### 2.2 Dataset construction and model training

#### 2.2.1 Data acquisition

**Figure 5:**
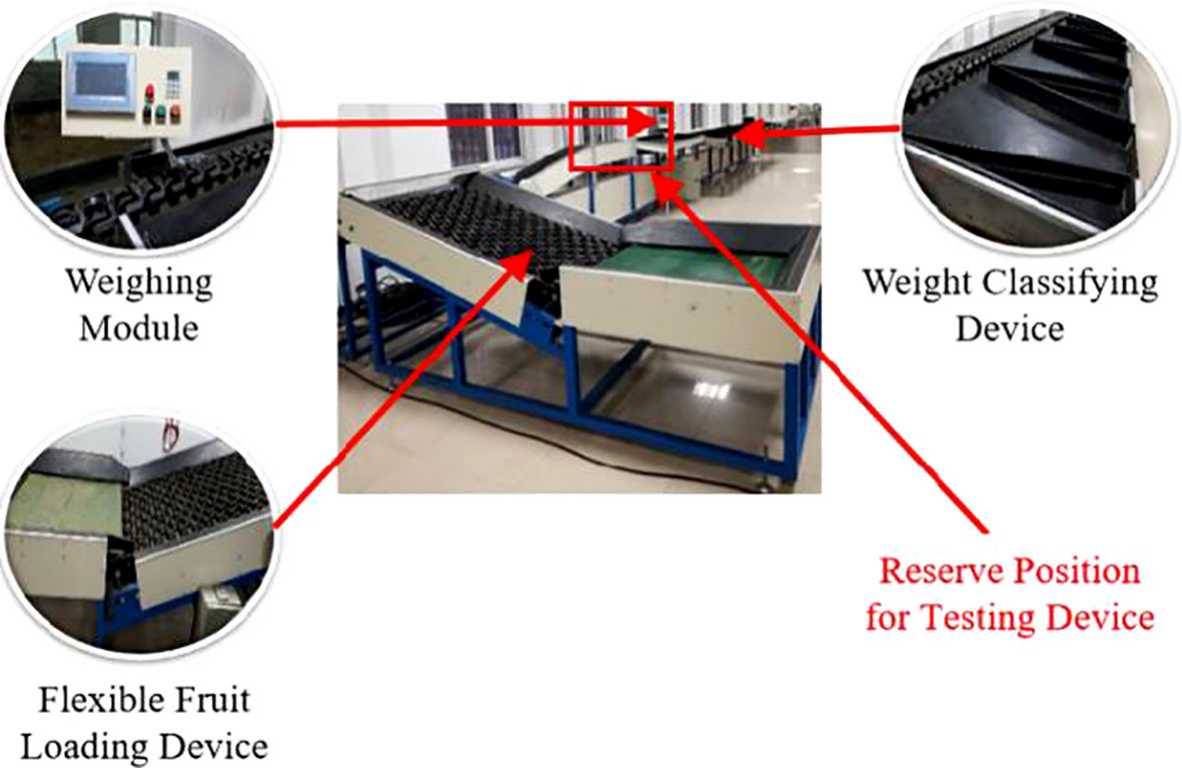
FGX-DZS type fruit & vegetable grading line.

In order to verify the effectiveness of the proposed method, industrial cameras were installed on the potato grading line to collect data, as illustrated in **Figure 5**.

**Figure 6:**
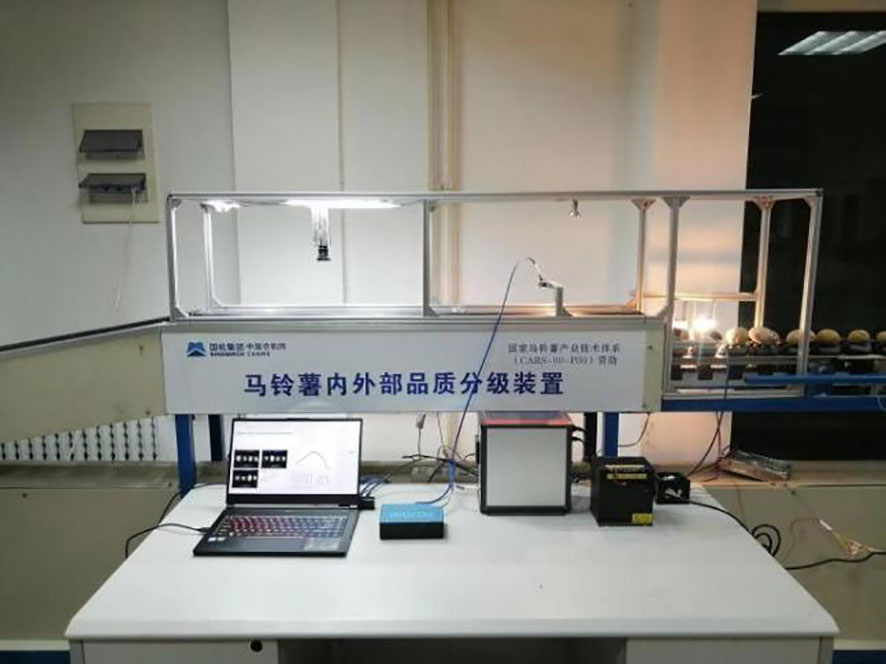
Acquisition scene diagram.

As shown in **Figure 6**, a CCD camera (model GS3-U3-15S5C-C from Point Grey) equipped with a LM5JCM lens from KOWA was used for image acquisition on the grading line, with the roller tray as the background. A 50W LED area light source was selected, and the camera, lens, and light source were arranged on the grading line. The light source height was adjusted to ensure that the images captured for various potato defects were neither overexposed nor reflective, with the final height set at 29 cm above the grading line. The camera parameters were set as follows: frame rate of 45 fps, image resolution of 1384×1032, and a pixel depth of 3×8 bit. A total of 1804 images were collected.

#### 2.2.2 Data processing

**Figure 7:**
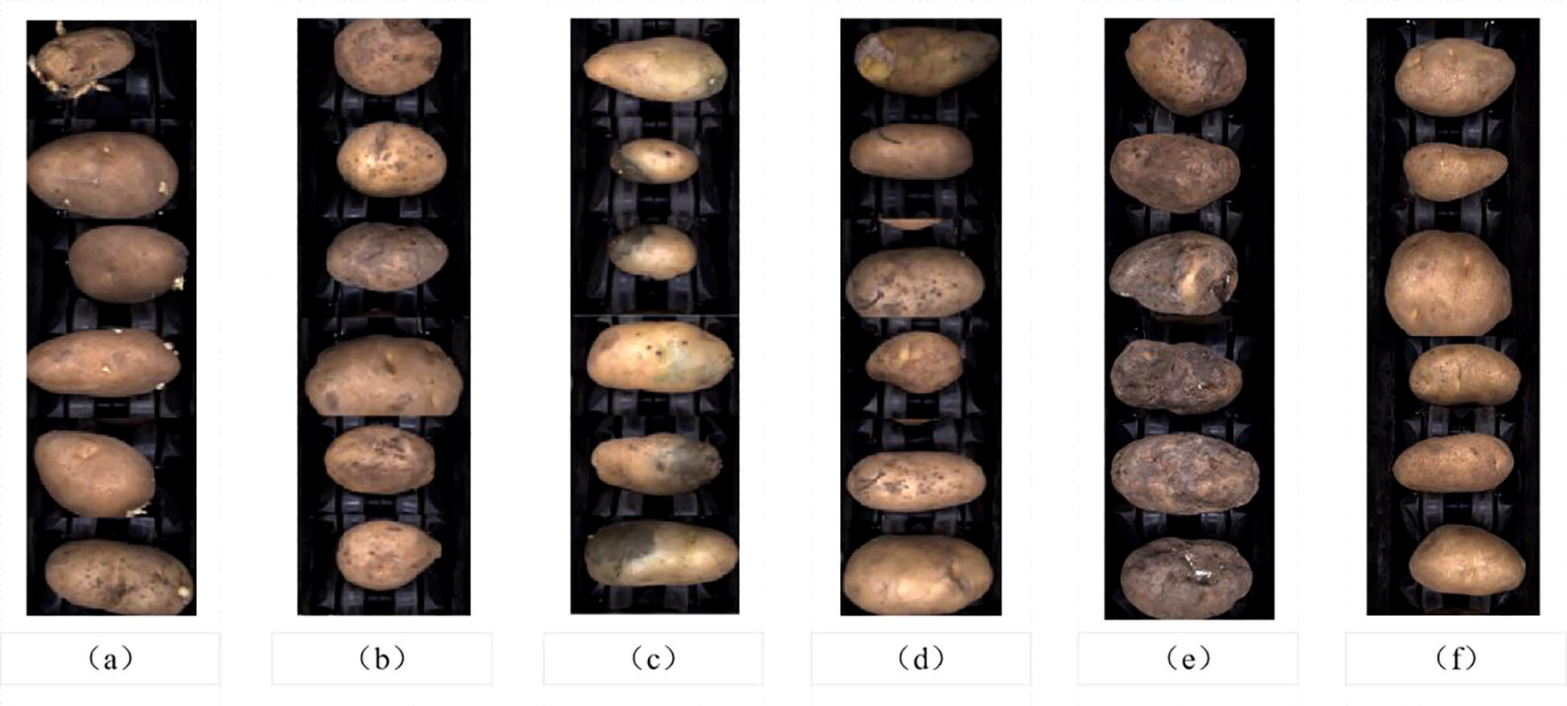
Classification of potato defects: **(A)** Sprouting. **(B)** Scab. **(C)** Green. **(D)** Damage. **(E)** Rot. **(F)** Health.

A total of 1804 collected images were annotated using the LabelImg tool, with categories including “health” “green” “sprouting” “scab” “rot” and “damage”. The annotation format was then converted as required. The original dataset was randomly divided into training (1082 images), validation (361 images), and test (361 images) sets in a 3:1:1 ratio, as shown in **Figure 7**. The distribution of each category within each dataset is detailed in **Table 1**.

**Table 1:** Partition of the dataset.

Categories	Number of train sets	Number of valid sets	Number of test sets
Health	238	77	75
Sprouting	192	64	63
Green	151	51	53
Scab	167	55	57
Rot	177	59	61
Damage	157	55	52

#### 2.2.3 Data enhancement

**Figure 8:**
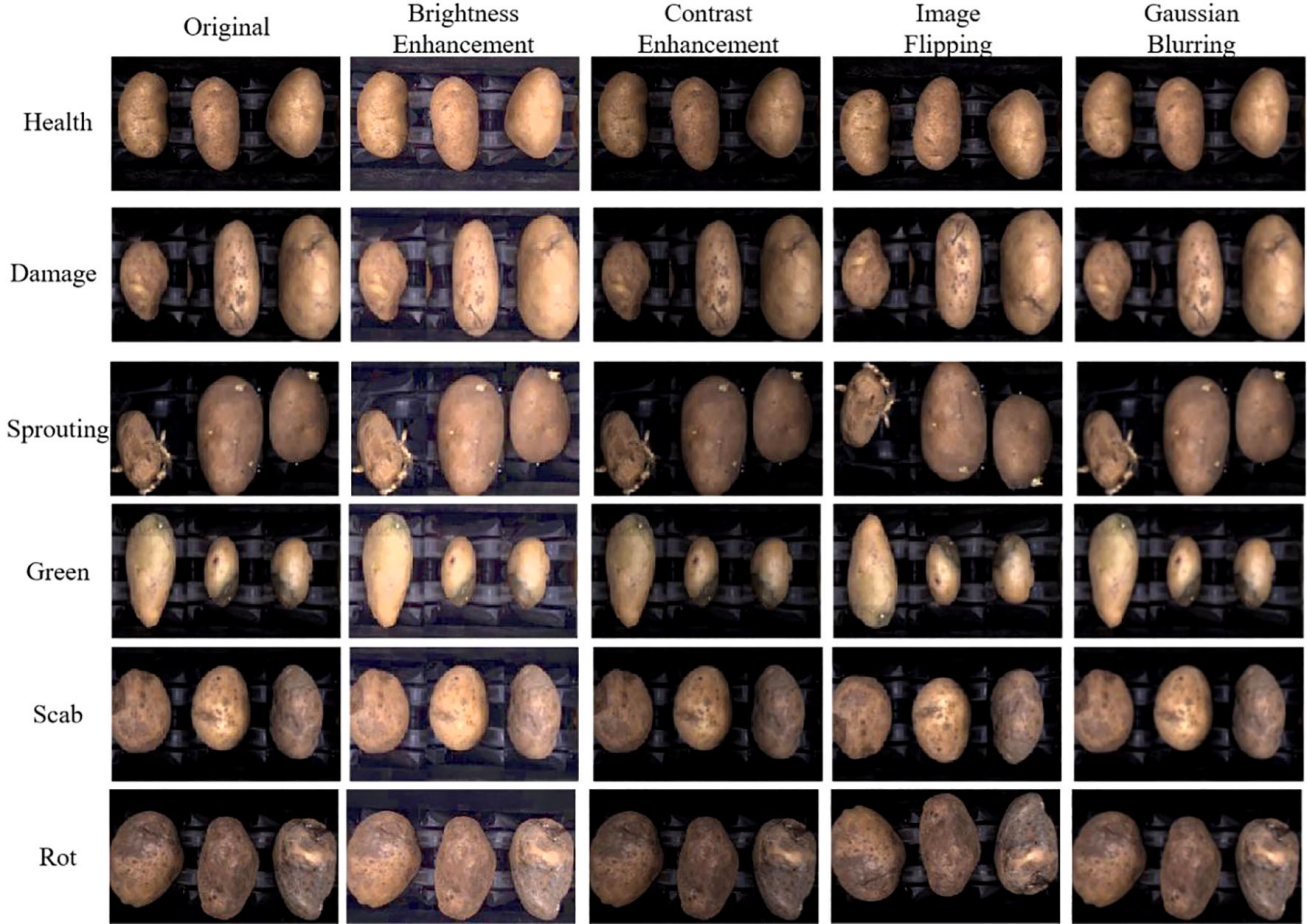
Potato data enhancement effect.

Data augmentation can enhance the model’s robustness. Insufficient or low-quality training samples can hinder the model’s generalization ability and robustness. To address this, several augmentation techniques were applied to the training set, including brightness enhancement, contrast enhancement, image flipping, and Gaussian blurring. Each augmentation method doubled the data, resulting in a total of 5410 images in the augmented training set. The effects of the image enhancement methods are shown in **Figure 8**.

#### 2.2.4 Experimental environment

The experiment was conducted on a Linux system, with Python as the programming language and Pycharm as the integrated development environment (IDE). Device specifications are listed in **Table 2**. The experiments utilized the SGD optimizer with a momentum of 0.937 and a weight decay of 0.0005. Training was performed over 100 epochs with a batch size of 16, an initial learning rate of 0.01, and a cosine decay learning rate strategy.

**Table 2:** Device configuration information.

Equipment Units/Software	Configuration
Central Processing Unit	Z7M-KP7GG Inter Core i7-8750H
Graphics Processing Unit	NVIDA GTX1050Ti 4GGDDR5
Operating Systems	Ubuntu18.04
Deep learning frameworks	Pytorch
Programming Languages	Python3.7

#### 2.2.5 Evaluation metrics

To evaluate the performance of the improved YOLO v5s model, Precision (P), Recall (R), F1-Score(F1) and Mean Average Precision (mAP) were selected as evaluation metrics. The relevant formulas are provided as follows:

Precision (P) measures the proportion of correctly identified positive samples among all samples with positive predictions, as shown in Equation 11:


(11) $$P=\frac{TP}{TP+FP}$$

Recall (R) measures the proportion of true positive samples that are correctly identified by the model, as shown in Equation 12.


(12) $$R=\frac{TP}{TP+FN}$$

F1-Score measures the harmonic mean of precision and recall, used to provide a comprehensive evaluation of a model’s performance in classification tasks, as shown in Equation 13.


(13) $$\text{F}1-\text{Score}=\frac{2P\times R}{P+R}$$

Mean average precision (mAP) is used to calculate the average precision (AP) across multiple categories, as shown in Equation 14. It involves averaging the precision values for each category, as defined in Equation 15:


(14) $$mAP=\frac{1}{n}\sum_{k=1}^{n}AP_{k}$$

(15) $$AP_{k}=\int_{0}^{1}prdr$$

Where *n* denotes the number of categories and *AP_k_* represents the accuracy (Average Precision) of the *k*-th category. The definitions of different prediction outcomes are as follows.:

True Positive (TP): The prediction is positive, the labeled value is positive, and the prediction is correct.

False Negative (FN): The prediction is negative, the labeled value is positive, and the prediction is incorrect.

False Positive (FP): The prediction is positive, the labeled value is negative, and the prediction is incorrect.

True Negative (TN): The prediction is negative, the labeled value is negative, and the prediction is correct.

## 3 Results and discussions

### 3.1 Performance comparison of different loss functions applied to YOLO v5s

**Figure 9:**
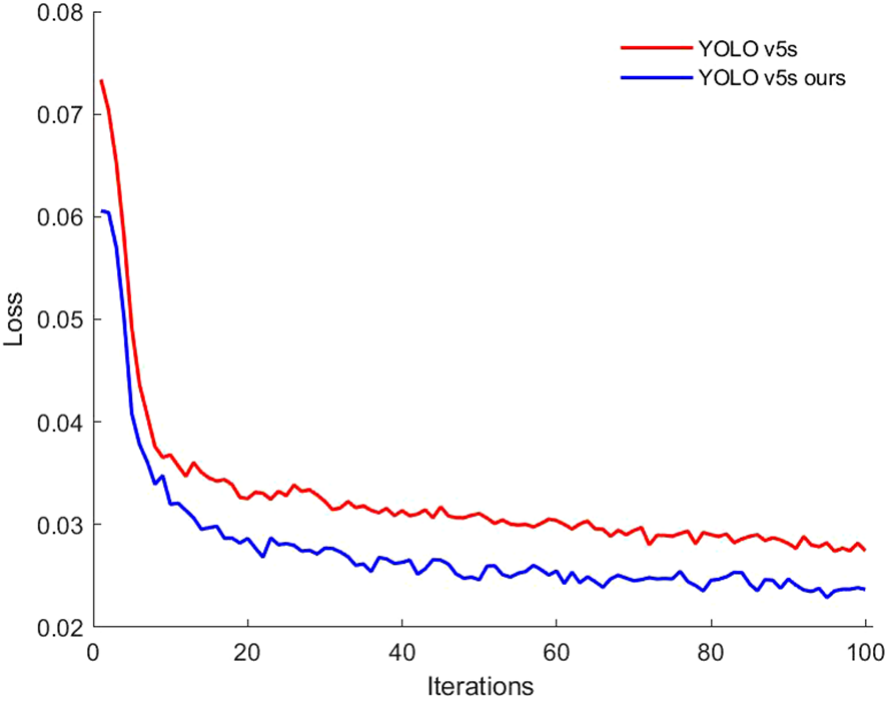
Comparison of regression losses for different loss functions' prediction frames.

To evaluate the impact of the loss function on model performance, the original YOLO v5s model was compared with a version that uses the Alpha-IoU loss function. The bounding box loss values during training were plotted as comparison curve to assess model performance, as shown in **Figure 9**. Both the improved model and the original YOLO v5s demonstrated a rapid decrease in loss during the first 10 iterations, indicating that the YOLO v5s model converged quickly. From the 11th to the 60th iteration, the loss values gradually decreased, stabilizing at 0.03 for the original model and 0.025 for the improved model, indicating good stability in the model’s performance. After 100 iterations, both models showed similar convergence trends, with loss values stabilizing. These results suggest that the Alpha-IoU loss function in the YOLO v5s model effectively reduces bounding box loss, thereby enhancing the detection model’s prediction capability.

### 3.2 Ablation experiments

To evaluate the effectiveness of the Coordinate attention (CA) module and the ASFF+ASPP structure in the proposed potato external defect detection model, an ablation study was conducted using the potato external defects dataset. The trained detection model was evaluated on the test set. The original YOLO v5s model served as the baseline, and three optimized models were created by incorporating the CA module and ASFF+ASPP structure: YOLO v5s-CA, YOLO v5s-AA, and YOLO v5s-ours (YOLO v5s+CA+ASFF+ASPP).

**Table 3** shows the P, R, F1-Score and mAP for each model across six categories: healthy, sprouting, greening, scabbing, rotting and mechanical damage. The results indicate that all the ablation models outperformed the original YOLO v5s in terms of detection accuracy. Specifically, the CA module led to improvements in P, R, F1-Score and mAP for the categories of healthy, sprouting, scabbing, rotting, and mechanical damage. For mechanical damage, P, R and F1-Score increased by 30.7%, 33.7% and 32.4%, respectively, while the other four categories saw improvements of approximately 0.5%-2%. Although the P, R, and F1-Score of greening decreased by 6.5%, 6.0%, and 6.2%, respectively, the mAP improved by 4.7%. These results suggest that CA improves model performance by capturing better global receptive fields and encoding precise location information.

**Table 3:** Ablation comparison trial results.

Model	Categories	P/%	R/%	F1-Score/%	mAP/%
YOLO v5s	Health	61.7	59.6	60.6	71.4
	Sprouting	69.5	68.3	68.8	
	Green	82.6	81.3	81.8	
	Scab	83.2	82.1	82.4	
	Rot	96.3	97.0	96.4	
	Damage	35.1	29.7	32.1	
YOLO v5s-CA	Health	62.9	60.8	61.7	76.1
	Sprouting	70.3	68.8	69.4	
	Green	76.1	75.3	75.6	
	Scab	84.5	83.9	84.4	
	Rot	97.0	97.3	97.0	
	Damage	65.8	63.4	64.5	
YOLO v5s-AA	Health	74.9	75.1	74.9	80.7
	Sprouting	82.0	81.3	81.6	
	Green	81.8	82.0	82.0	
	Scab	87.5	86.3	86.6	
	Rot	97.0	97.3	97.0	
	Damage	60.7	59.9	60.2	
YOLO v5s-ours	Health	77.0	75.6	76.1	85.1
	Sprouting	86.0	84.5	85.2	
	Green	87.1	86.2	86.5	
	Scab	89.6	88.3	88.9	
	Rot	96.3	97.0	96.4	
	Damage	74.8	74.2	74.5	

The ASFF+ASPP structure enhanced the P and R for healthy, sprouting, scabbing, and mechanical damage by 13.2%, 12.3%, 4.3%, 25.6%, respectively, and 15.5%, 13%, 4.2%, 30.2%, in recall. Although P and R for greening decreased by about 1%, and mAP improved by 9.3%. The F1-score of the six categories increased by 14.3%, 12.8%, 0.2%, 4.2%, 0.6%, and 28.1%, respectively. This demonstrates that the ASFF+ASPP structure improves model performance through weighted fusion of features, enhanced receptive fields, and multi-scale features extraction.

The integration of both CA and ASFF+ASPP (YOLO v5s-ours) resulted in a substantial improvement in P and R across all categories, with increases of 15.3%, 16.5%, 4.5%, 6.4%, 39.7% and 16.0%, 16.2%, 4.9%, 6.2%, 44.3%, respectively, for healthy, sprouting, greening, scabbing, and mechanical damage. The F1 scores increased by 15.5%, 16.4%, 4.7%, 6.5%, and 42.4%, respectively. There was no change in the metrics for the rotting category, and the mAP increased by 13.7%. The combination of CA and ASFF+ASPP significantly boosted the model’s accuracy in detecting surface defects on potatoes, enabling more precise localization and feature extraction, which reduces inspection bias.

**Figure 10:**
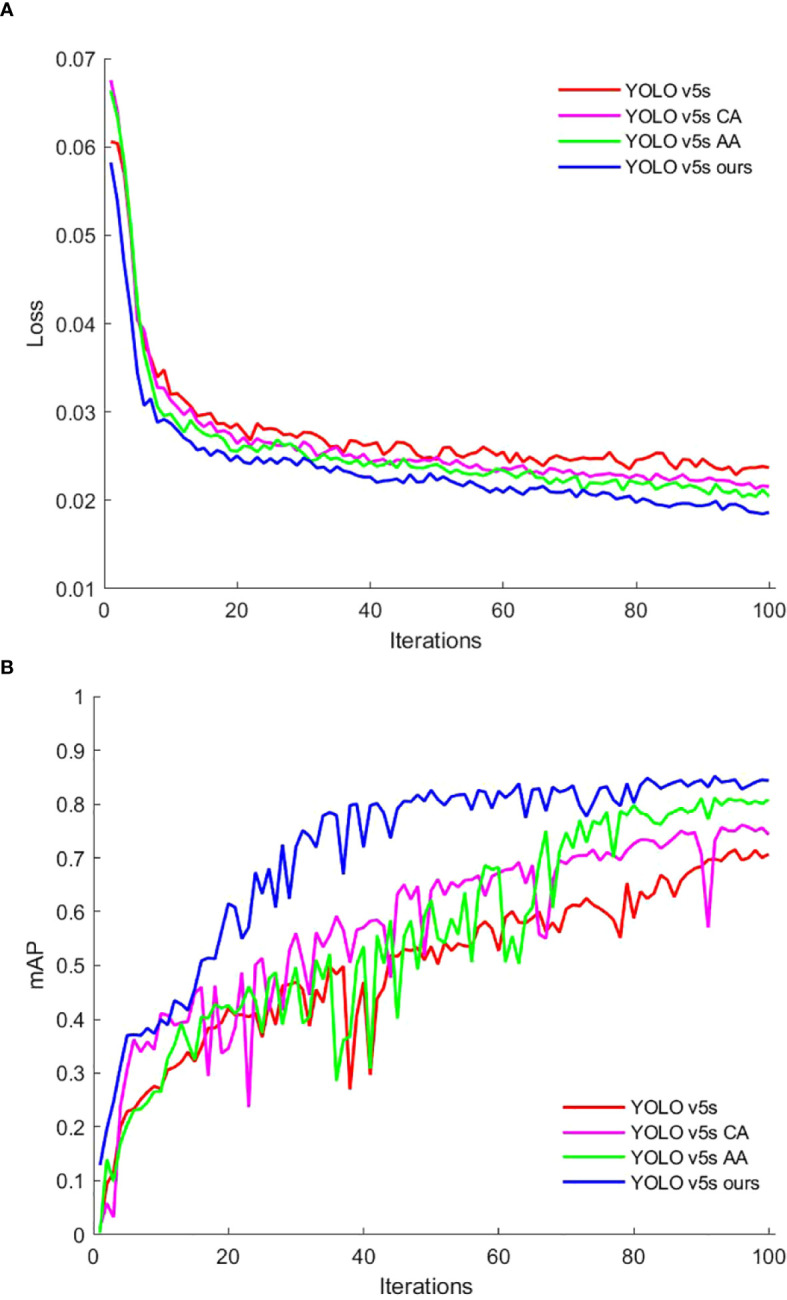
Evaluation metrics change curves: **(A)** Train set loss. **(B)** Test set mAP.

**Figure 10** illustrates the loss curves on the training set and the mAP on the test set for each model in the ablation test, plotted against the number of iterations.

**Figure 11:**
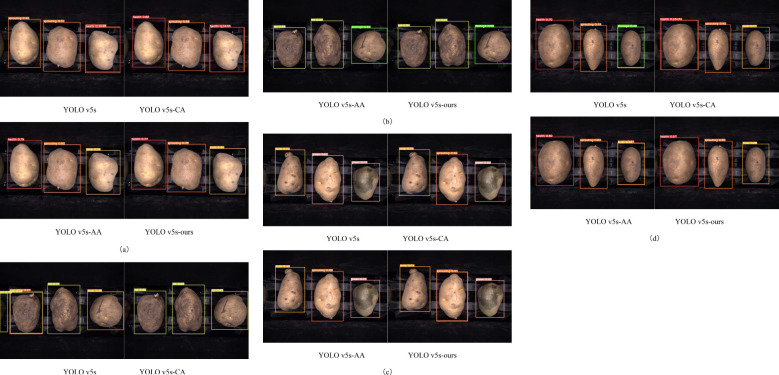
Recognition Performance of Potatoes in Each Category Across Four Models: **(A)** Healthy, Sprouting, Scab. **(B)** Rot, Rot, Damage. **(C)** Scab, Sprouting, Greening. **(D)** Healthy, Sprouting, Scab.

**Figure 11** shows the classification results of each model on the test set from the ablation study. It is evident that the introduction of the CA module and the ASFF+ASPP architecture has played a significant role in improving the model’s performance. The CA module significantly improves the detection performance for the healthy, sprouting, and rot categories, particularly enhancing the recognition of healthy and rot defects. On the other hand, the ASFF+ASPP architecture excels in multi-scale feature extraction, especially improving the detection of sprouting, scab, and damage categories, enabling better capture of defects at varying scales. The YOLO v5s-ours model, by integrating both the CA module and the ASFF+ASPP structure, achieves the best overall performance, with near-perfect results in the detection of rot defects.

### 3.3 Model performance comparison

To further validate the effectiveness of the proposed YOLO v5s model for detecting external defects in potatoes, comparisons were made with other representative target detection models, including Faster R-CNN, YOLO v6, YOLOX, and YOLO v7. To ensure a fair comparison, all models were trained using the same strategy and computational environment. Performance was evaluated using P, R, F1-Score and mAP.

**Table 4** presents the performance metrics of each model on the test set. The results indicate that the original YOLO v5s model outperforms other YOLO series models in terms of overall performance, supporting its selection as the base model for further optimization in this study. Among the models tested, the proposed YOLO v5s-ours model demonstrates the best accuracy in detecting potato surface defects. This improvement is attributed to the CA attention mechanism, which focuses the network on defect regions, and the use of atrous convolutions with varying dilation rates, which capture defect information at different scales. Additionally, the adaptive feature fusion strategy enhances the network’s feature extraction capabilities, thereby improving detection accuracy for potato surface defects.

**Table 4:** Comparison of the effect of different models.

Model	P/%	R/%	F1-Score/%	mAP/%	Memory Usage/MB	Frame Rate/(f·s^-1^)
Faster R-CNN	56.7	76.9	65.1	69.1	325.8	14.3
YOLO v6	52.6	74.8	61.7	65.9	34.3	28.3
YOLOX	56.3	77.0	65.1	70.8	54.2	23.1
YOLO v7	54.5	76.7	63.7	69.4	36.9	12.8
YOLO v5s	57.4	75.1	64.9	71.4	13.7	31.9
YOLO v5s-ours	82.0	86.6	84.3	85.1	23.3	30.7

The YOLO v5s model shows the second-highest recognition accuracy after YOLO v5s-ours, achieving a P, R, F1-Score and mAP of 57.4%, 75.1%, 64.9% and 71.4%, respectively. In contrast, YOLO v6 exhibits the lowest recognition accuracy, with a P, R, F1-Score and mAP of 52.6%, 74.8%, 61.7% and 65.9%, respectively. The mAP of YOLO v5s-ours reaches 85.1%, which is 19.2% higher than YOLO v6 and 13.7% higher than the original YOLO v5s model.

The addition of the CA, ASFF, and ASPP modules increases the model’s memory usage from 13.7 MB to 23.3 MB, while the detection frame rate decreases slightly from 31.9f/s to 30.7f/s. Despite these changes, the minor reduction in frame rate and the increase in memory usage do not hinder practical application. Therefore, the proposed model maintains high mAP and frame rate, balancing detection accuracy and recall rate, achieving optimal overall performance.

### 3.4 Potato grading line external defect detection test

To assess the effectiveness of the improved YOLO v5s model for detecting external defects on an operational potato grading line, tests were conducted using an FGX-DZS type fruit and vegetable grading line. Various categories of potatoes were positioned under a CCD camera, which captured images of the potatoes. These images were then processed by the model on a computer to detect external defects. The sample sizes for each category—healthy, sprouting, greening, scab, rot, and mechanical damage—were 50, 20, 20, 20, 20 and 20, respectively. The results of the tests are summarized in **Table 5**.

**Table 5:** Potato grading line external defect detection results.

Model	Categories	Identify Correct Sample	Identify Error Sample	Accuracy/%
YOLO v5s	Health	32	18	64
	Sprouting	16	4	80
	Green	15	5	75
	Scab	17	3	85
	Rot	20	0	100
	Damage	7	13	35
YOLO v5s-ours	Health	40	10	80
	Sprouting	16	4	80
	Green	18	2	90
	Scab	18	2	90
	Rot	20	0	100
	Damage	12	8	60

The findings indicate a marked improvement in the model’s recognition rates, particularly for healthy potatoes and those with mechanical damage, in real-world detection applications. This suggests that the improved YOLO v5s model developed in this study effectively reduces the false detection rate in practical grading line operations.

**Figure 12:**
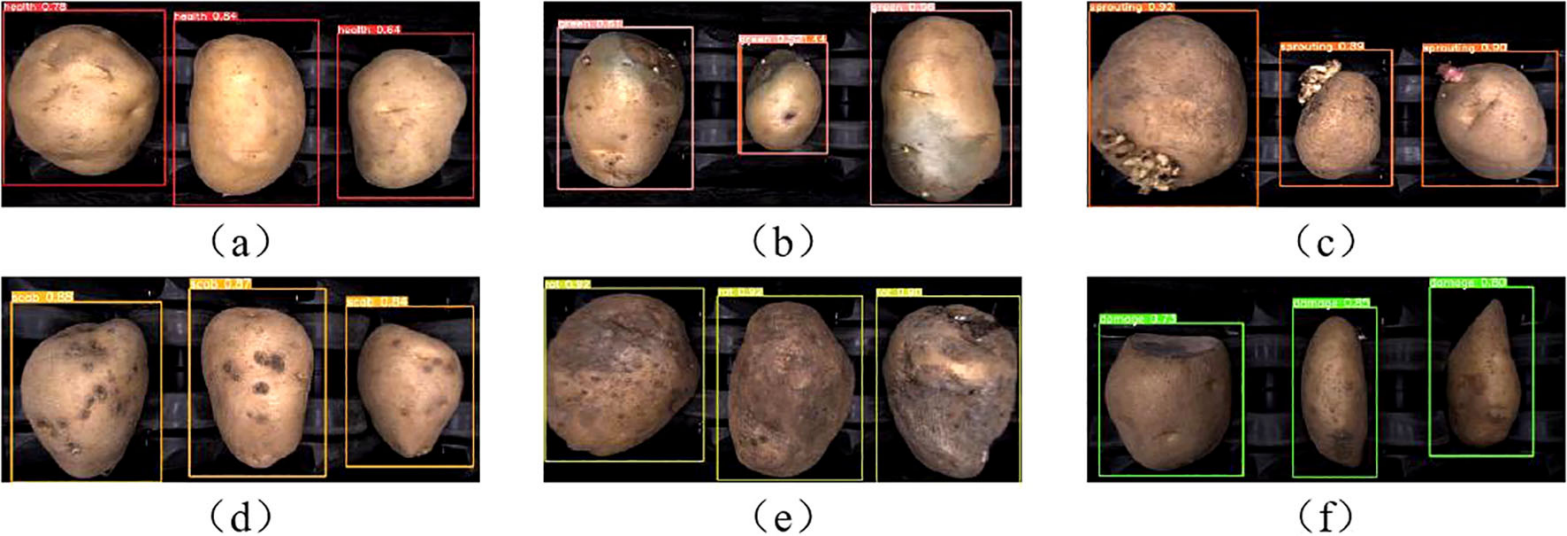
Identify correct sample: **(A)** Health. **(B)** Green. **(C)** Sprouting. **(D)** Scab. **(E)** Rot. **(F)** Damage.

**Figure 13:**
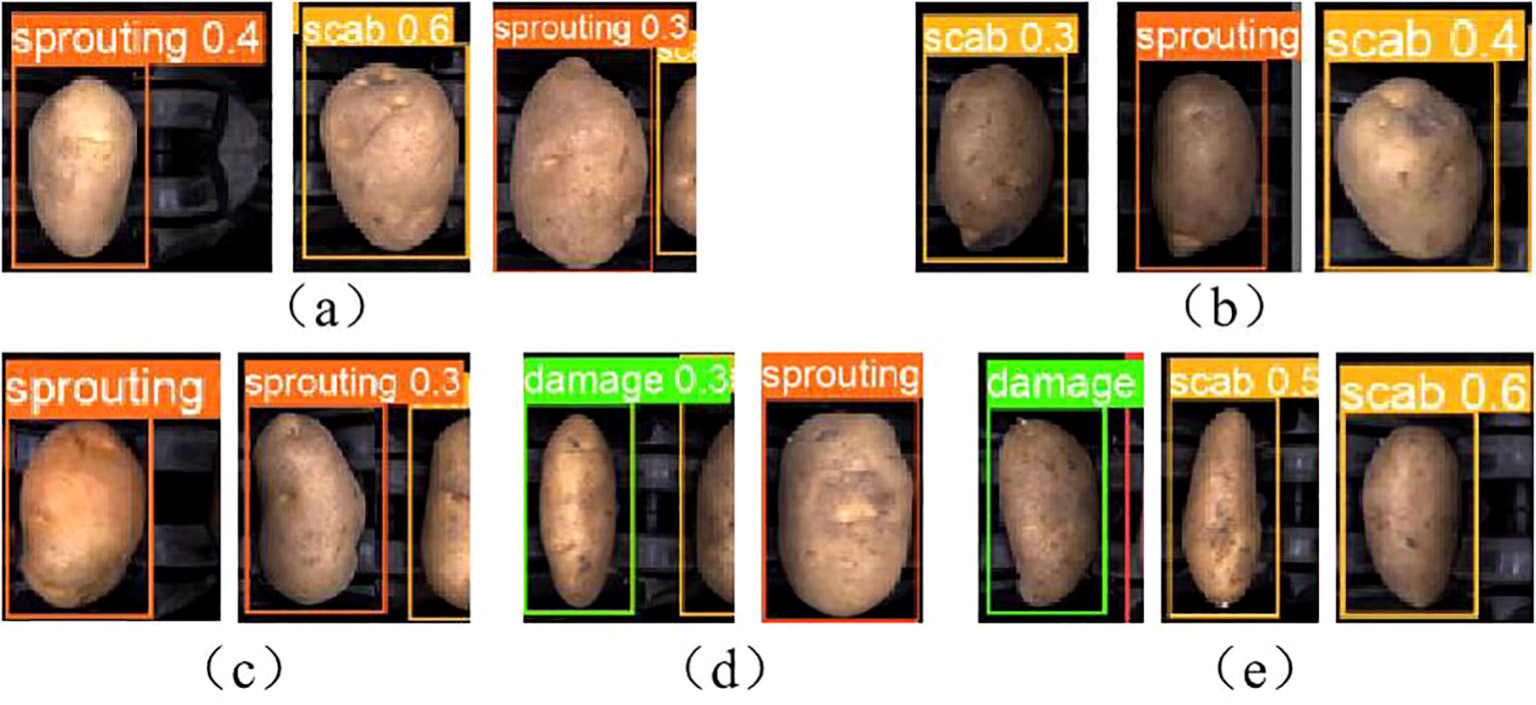
Identify error sample: **(A)** Health misclassification. **(B)** Damage misclassification. **(C)** Green misclassification. **(D)** Scab misclassification. **(E)** Sprouting misclassification.

**Figures 12** and **13** illustrate correctly and incorrectly recognized samples by the original model, respectively. It can be observed that the model accurately identifies defects when they are prominent, but also exhibits varying degrees of misclassification.

In **Figure 13A**, a healthy potato has a small depression on its surface. This minor feature may have been extracted during the convolution process and misinterpreted as characteristics of sprouting or scab. In **Figure 13B**, the damaged area of a mechanically damaged potato is located on the side, making it difficult for the camera to capture accurately from a top-down perspective. Surface depressions and residual soil also interfere with identification, resulting in misclassification as sprouting or scab.

**Figure 13C** shows a greening potato with a green area along the edge, appearing darker in color. Depressions on the surface contribute to its misidentification as a sprouted potato. In **Figure 13D**, dark spots on a scabbed potato are misinterpreted as mechanical damage, while surface depressions are identified as sprouting. In **Figure 13E**, a sprouted potato with small sprouts located on the side is misclassified due to surface soil and the potato’s irregular shape, resulting in identification as scab and mechanical damage.

**Figure 14:**
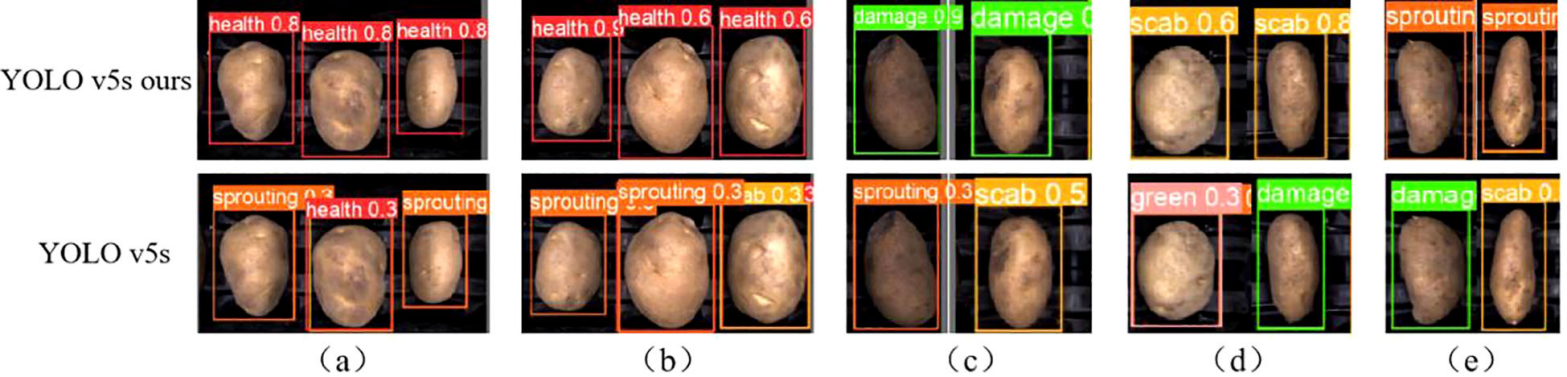
Comparison of practical application testing: **(A)** Health. **(B)** Health. **(C)** Damage. **(D)** Scab. **(E)** Sprouting.

**Figure 14** presents a comparison between the detection results of the improved YOLO v5s (ours) and the original YOLO v5s model. The improved model successfully identifies defects correctly, whereas the original model misclassifies them. In **Figures 14A** and **B**, the improved model effectively reduces misclassification of healthy yams with surface depressions. This improvement suggests that the Coordinate Attention (CA) module, positioned at the front of the detection head, enhances weight assignment to the depression feature, allowing it to differentiate depression from defects like scab and sprouting.

In **Figures 14C** and **E**, the mechanical damage and sprouting are located on the edge of the potato body, causing misclassification in the original model. The ASFF+ASPP (Adaptive Spatial Feature Fusion and Atrous Spatial Pyramid Pooling) structure in the improved model expands the receptive field, incorporating more original features during downsampling to enrich defect information. Furthermore, ASFF’s feature fusion mechanism assigns greater weight to defective features, preserving feature information and improving the model’s detection of small, edge-located features.

These results demonstrate that the improved YOLO v5s model developed in this study is suitable for detecting external defects in potatoes.

## 4 Conclusions

1. To enable accurate detection of external defects in potatoes, this study proposes an efficient, precise, and highly adaptable improved YOLO v5s-based detection method, providing important technical support for the development of automated potato sorting systems. The introduction of the CA module enhances the model’s ability to handle subtle defects in potatoes. The ASFF+ASPP structure expands the receptive field and improves feature fusion, while assigning different weights to multi-scale feature maps, thereby enhancing the model’s inference ability for cross-scale feature fusion. This addresses the issue of multi-scale object detection, enabling the model to capture richer contextual information. Additionally, replacing the traditional loss function with the Alpha-IoU function accelerates model convergence and further enhances detection performance for potato external defects.
2. The experimental results demonstrate that the precision, recall, F1-Score and mAP of the improved YOLO v5s model are 82.0%, 86.6%, 84.3%, and 85.1%, respectively. Compared to the original model, the improved model achieves significant gains: precision, recall, and F1-Score for healthy potatoes, germination, greening, scab and mechanical damage were enhanced by 15.3%, 16.5%, 4.5%, 6.4%, 39.7% and 16.0%, 16.2%, 4.9%, 6.2%, 44.3% and 15.5%, 16.4%, 4.7%, 6.5%, 42.4%, respectively, with mAP increasing by 13.7%. A comparative analysis of different models shows that the improved YOLO v5s model outperforms Faster R-CNN, YOLO v6, YOLOX, and YOLO v7, achieving mean average precision improvements of 16.0%, 19.2%, 14.3%, 15.7%, and 13.7%, respectively.
3. The potato grading line external defect detection test further validates the strong performance of the model. This provides a novel approach for non-destructive testing of external defects in potatoes. However, some areas still require improvement: ① The roller tray of the grading line is black, which can resemble certain potato defects, thus affecting detection accuracy. ② To improve accuracy, adding more cameras, particularly positioned on the sides, would enable better capture of potato side images.

## Data Availability

The original contributions presented in the study are included in the article/supplementary material. Further inquiries can be directed to the corresponding authors.

## References

[B1] Bochkovskiy A. Wang C. Y. Liao H. Y. M. (2020). “YOLOv4: optimal speed and accuracy of object detection,” in 2020 IEEE Conference on Computer Vision and Pattern Recognition (CVPR), Seattle, WA, USA. doi: 10.48550/arXiv.2004.10934

[B2] Cui Y. W. Du C. H. Li S. J. (2020). Analysis and prospect on China’s seed potato industrial development. Agric. Outlook. 16, 71–76. doi: 10.3969/j.issn.1673-3908.2020.01.015

[B3] Fu Y. L. Liang D. Liang D. T. Fang N. Cheng X. E. (2021). Potato surface defect detection based on machine vision and YOLO algorithm. Machinery 59, 82–87. doi: 10.3969/j.issn.1000-4998.2021.08.022

[B4] Ge Z. Liu S. T. Wang F. Li Z. M. Sun J. (2021). “YOLOX: exceeding YOLO series in 2021,” in 2021 IEEE Conference on Computer Vision and Pattern Recognition (CVPR), Nashville, TN, USA. doi: 10.48550/arXiv.2107.08430

[B5] He B. Zhang Y. B. Gong J. L. Fu G. Zhao Y. Q. Wu R. D. (2022). Fast recognition of tomato fruit in greenhouse at night based on improved YOLO v5. Trans. Chin. Soc. Agric. Machine. 53, 201–208. doi: 10.6041/j.issn.1000-1298.2022.05.020

[B6] Li S. P. Zheng C. R. Wen C. M. Li K. H. Gan W. G. Li Y. (2023). Stem node feature recognition and positioning technology for transverse cutting of sugarcane based on improved YOLO v5s. Trans. Chin. Soc. Agric. Machine. 54, 234–245 + 293. doi: 10.6041/j.issn.1000-1298.2023.10.023

[B7] Li Y. H. Li T. H. Niu Z. R. Wu Y. Q. Zhang Z. L. Hou J. L. (2018). Potato dud eyes recognition based on three-dimensional geometric features of color saturation. Trans. Chin. Soc. Agric. Eng. 34, 158–164. doi: 10.11975/j.issn.1002-6819.2018.24.019

[B8] Luo Q. Y. Lun R. Q. Gao M. J. Liu Y. (2022). Strategy path of high-quality development of potato industry in China during from 2021 to 2025. Chin. J. Agric. Resour. Reg. Plan. 43, 37–45. doi: 10.7621/cjarrp.1005-9121.20220305

[B9] Lv Z. Q. Qi X. T. Zhang W. Z. Liu Z. D. Zheng W. X. (2021). Mu G.Z. Buds recognition of potato images based on Gabor feature. J. Agric. Mechan. Res. 43, 203–207. doi: 10.13427/j.cnki.njyi.2021.02.036

[B10] Redmon J. Farhadi A. (2018). “YOLOv3: an incremental improvement,” in 2018 IEEE Conference on Computer Vision and Pattern Recognition (CVPR), Salt Lake City, UT, USA. doi: 10.48550/arXiv.1804.02767

[B11] Ren S. Q. He K. M. Girshick R. Sun J. (2017). Faster R-CNN: towards real-time object detection with region proposal networks. IEEE Trans. Pattern Anal. Mach. Intell. 39, 1137–1149. doi: 10.1109/TPAMI.2016.2577031 27295650

[B12] Senthil V. K. Jaganathan M. Viswanathan A. Umamaheswari M. Vignesh J. (2023). Rice leaf disease detection based on bidirectional feature attention pyramid network with YOLO v5 model. Environ. Res. Commun. 5, 065014. doi: 10.1088/2515-7620/acdece

[B13] Shi F. Q. Wang H. L. Huang H. (2022). Research on potato buds detection and recognition based on convolutional neural network. J. Chin. Agric. Mechan. 43, 159–165. doi: 10.13733/j.jcam.issn.20955553.2022.06.021

[B14] Wang Y. Yao X. Z. Li B. Xu S. Yi Z. F. Zhao J. H. (2023). Malformed sweet pepper fruit identification algorithm based on improved YOLO v7-tiny. Trans. Chin. Soc. Agric. Machine. 54, 236–246. doi: 10.6041/j.issn.1000-1298.2023.11.023

[B15] Yang Y. Liu Z. F. Huang M. Zhu Q. B. Zhao X. (2022). Automatic detection of multi-type defects on potatoes using multispectral imaging combined with a deep learning model. J. Food Engin. 226, 111213. doi: 10.1016/j.jfoodeng.2022.111213

[B16] Zhang W. Z. Zeng X. Liu S. F. Mu G. Z. Zhang H. Y. Guo Z. Z. (2023). Detection method of potato seed bud eye based on improved YOLO v5s. Trans. Chin. Soc. Agric. Machine. 54, 260–269. doi: 10.6041/j.issn.1000-1298.2023.09.026

[B17] Zhang Z. G. Zhang Z. D. Li J. N. Wang H. Y. Li Y. B. Li D. H. (2021). Potato detection in complex environment based on improved YoloV4 model. Trans. Chin. Soc. Agric. Eng. 37, 170–178. doi: 10.11975/j.issn.1002-6819.2021.22.019

[B18] Zhao P. H. Meng C. N. Chang S. J. (2019). Single shot multibox detector based on asynchronous convolution factorization and shunt structure. J. Beijing Univ. Aeronaut. Astronaut. 45, 2089. doi: 10.13700/j.bh.1001-5965.2018.0564

[B19] Zhu X. K. Lyu S. Wang X. Xu S. Zhao Q. (2021). “TPH-YOLOv5: improved YOLOv5 based on transformer prediction head for object detection on drone-captured scenarios,” in 2021 IEEE/CVF International Conference on Computer Vision Workshops (ICCVW), Montreal, BC, Canada. 2778–2788. doi: 10.1109/ICCVW54120.2021.00312

